# Prefrontal cortex oxygenation during a mentally fatiguing task in normoxia and hypoxia

**DOI:** 10.1007/s00221-024-06867-y

**Published:** 2024-06-05

**Authors:** Jonas De Wachter, Manon Roose, Matthias Proost, Jelle Habay, Matthias Verstraelen, Sander De Bock, Kevin De Pauw, Romain Meeusen, Jeroen Van Cutsem, Bart Roelands

**Affiliations:** 1 Human Physiology and Sports Physiotherapy Research Group, Vrije Universiteit Brussel, Brussels, Belgium; 2 Department of Radiology, UZ Brussel, Brussels, Belgium; 3 Research Foundation Flanders (FWO), Brussels, Belgium; 4 BruBotics, Vrije Universiteit Brussel, Brussels, Belgium; 5 VIPER Research Unit, Royal Military Academy, Brussels, Belgium

**Keywords:** Cognitive fatigue, Brain oxygenation, Functional near infrared spectroscopy, Hypoxia, Normoxia

## Abstract

Mental fatigue (MF) and hypoxia impair cognitive performance through changes in brain hemodynamics. We want to elucidate the role of prefrontal cortex (PFC)-oxygenation in MF. Twelve participants (22.9 ± 3.5 years) completed four experimental trials, (1) MF in (normobaric) hypoxia (MF_HYP) (3.800 m; 13.5%O_2_), (2) MF in normoxia (MF_NOR) (98 m; 21.0%O_2_), (3) Control task in HYP (CON_HYP), (4) Control in NOR (CON_NOR). Participants performed a 2-back task, Digit Symbol Substitution test and Psychomotor Vigilance task before and after a 60-min Stroop task or an emotionally neutral documentary. Brain oxygenation was measured through functional Near Infrared Spectroscopy. Subjective feelings of MF and physiological measures (heart rate, oxygen saturation, blood glucose and hemoglobin) were recorded. The Stroop task resulted in increased subjective feelings of MF compared to watching the documentary. 2-back accuracy was lower post task compared to pre task in MF_NOR and CON_NOR, while no differences were found in the other cognitive tasks. The fraction of inspired oxygen did not impact feelings of MF. Although performing the Stroop resulted in higher subjective feelings of MF, hypoxia had no effect on the severity of self-reported MF. Additionally, this study could not provide evidence for a role of oxygenation of the PFC in the build-up of MF.

**Supplementary Information:**

The online version contains supplementary material available at 10.1007/s00221-024-06867-y.

## Introduction

Mental fatigue is a psychobiological state characterized by sensations of tiredness and diminished energy levels resulting from high cognitive load (e.g. prolonged cognitive activity with low complexity/difficulty or short-time cognitive activity with high complexity/difficulty) (Borragán et al. 2017; Staiano et al.^ 2023^; Van Cutsem et al. 2017) and is known to impair multiple aspects of cognitive performance (Boksem and Tops 2008; Lorist et al. 2000; Pageaux and Lepers 2018; Pageaux et al. 2014; Russell et al. 2019; Van Cutsem et al. 2019a). In general, cognitive performance is most easily impaired by mental fatigue if it relies on executive functions (i.e. higher-order cognitive control processes for the attainment of a specific goal), while more automatic cognitive processing is relatively insensitive to this state (Fisk and Scerbo 1987; Van Cutsem et al. 2019b; van der Linden et al. 2003a, b; van der Linden et al. 2003a, b). In laboratory settings, mental fatigue can be assessed through standardized tasks designed to evaluate cognitive performance under conditions of cognitive strain or prolonged mental exertion. The Psychomotor Vigilance Test (PVT) is a prime example of such a task. The PVT involves participants responding to visual stimuli with rapid and accurate reactions over a sustained period, typically ranging from several minutes to half an hour (Honn et al. 2015). As mental fatigue accumulates during the task, performance indicators such as reaction time and accuracy tend to decrease. These decrements in performance serve as objective markers of mental fatigue, allowing quantitative analysis to evaluate the impact of cognitive strain (Smith et al. 2019). Recently, it has been shown that these mental fatigue-induced impairments in cognitive performance also persist in more sport-specific settings, since mental fatigue is known to negatively impact endurance performance, mainly through increased perceived exertion (Van Cutsem et al. 2017). Additionally it was recently stated that mental fatigue also impairs Sport-Specific Psychomotor Performance (Habay et al. 2021). An interest has arisen to gain further insight into the mechanism behind these mental fatigue associated impairments in cognitive performance, both in a sport-specific and non-sport-specific setting (Pattyn et al. 2018; Van Cutsem et al. 2022).

Since the PFC plays an important role in the executive functions impaired by mental fatigue (Van Cutsem et al. 2017), it is suggested that the PFC functioning affects the mental fatigue-induced impairments in cognitive performance (Boksem and Tops 2008; Boksem et al. 2006; van der Linden and Eling 2006; Pageaux and Lepers 2018; Smith et al. 2019, 2016). A possible hypothesis is that a decrease in regional cerebral oxygenation, which may be induced by mental fatigue, can alter the functioning of the PFC. The hemodynamics within this brain region can be measured by using prefrontal functional Near Infrared Spectroscopy (fNIRS), a non-invasive way of measuring changes in oxygenated- and deoxygenated hemoglobin in the cerebral blood flow (CBF). fNIRS has already been used for measuring cerebral oxygenation at the PFC in cognitive research. These studies described increases in oxygenated hemoglobin (HbO_2_) and decreases in deoxygenated hemoglobin (HHb) during the execution of cognitive tasks, compared to baseline (Angius et al. 2022; Causse et al. 2017a, b; Kaneko et al. 2011; Mehta, and Parasuraman 2014; Vermeij et al. 2017). This assumption of decreased oxygenation specifically due to mental fatigue has already been explored by Li et al. (2009) by using a prolonged simulated driving task. The authors observed an increased frontal cortex oxygenation at the beginning of the driving task, followed by a decreased oxygenation near the end. Li et al. (2009) proposed that this initial increase might be attributed to the increased cerebral blood flow in response to the brain’s energy demands. This finding underlines the value of prefrontal fNIRS in research aiming at further investigating mechanisms of mental fatigue.

Another stressor that also impairs cognitive performance is hypoxia (McMorris et al. 2017). A recent meta-analysis of McMorris et al. (2017) indicated that low (< 60 mmHg) arterial partial pressure of O_2_ (PaO_2_) levels resulted in a deterioration in, among other functions, attention, response inhibition and working memory. This is corroborated by two reviews (Taylor et al. 2015; Virues-Ortega et al. 2004) that described the detrimental effects of exposure to altitude on cognitive functioning in simple and complex tasks. Hypoxic conditions in these trials resulted in a slower reaction times (Davranche et al. 2016; Phillips et al. 2015; Taylor et al. 2015), decreased accuracy (Davranche et al. 2016), impaired concentration (Turner et al. 2015), deficits in learning and (short term- and spatial) memory (Wilson et al. 2009a). However, Geissler et al. (2023) demonstrated that the impact of an acute stressor on working memory is non-linear, with effects evolving over time.The mechanisms behind these decreases are still not fully elucidated yet (Davranche et al. 2016; McMorris et al. 2017). Obviously, acute hypoxia will reduce the slope of the oxygen transport cascade from the atmosphere to the mitochondria, resulting in a lower PaO_2_ and reduced oxyhemoglobin saturation. Subsequently, the initial response of the human body to acute hypoxia (i.e. increase in peripheral adrenaline levels, heart rate and cardiac output and a decrease in plasma volume) aims to maintain oxygen supply (McMorris et al. 2017). This initial response seems however to be unable to uphold cognitive performance in a hypoxic environment (McMorris et al. 2017; Wilson et al. 2009b). In various real-world contexts, hypoxia and mental fatigue occur simultaneously, including high-altitude trekking, aircraft piloting, and space missions (Hu and Lodewijks 2020).

Changed cognitive functioning at altitude is also associated with alterations in brain oxygenation (Phillips et al. 2015). A decrease in cerebral oxygen saturation, an increased CBF and a shift in HbO_2_ and HHb (Davranche et al. 2016) are typical hemodynamic responses during prolonged cognitive activity. In theory, these hemodynamic responses are preferably measured at the PFC, which is known for its role in higher-level cognitive function and decision making (Bechara et al. 1999; Krawczyk 2002), planning, attention and (short-term) memory. However, research shows that there are challenges to observe these hemodynamic changes. Decroix et al. (2018), Davranche et al. (2016) Lefferts et al. (2016) reported that acute hypoxia did not change the HbO_2_ due to local cerebral vasodilatation during cognitive tasks. These studies used prefrontal fNIRS, but oxygen saturation (SaO_2_) was measured at the index finger, indicating peripheral- and not central oxygenation. Phillips et al. (2015), however, were able to show that the observed drop in arterial oxygen saturation at the index finger was also present in the PFC. Phillips et al. (2015) further supported the previous findings in terms of cognitive functioning, with slower simple- and choice reaction times and negatively altered aspects of visual processing. Given these contradictory findings in literature, it is clear that further research into prefrontal oxygenation is necessary to further elucidate impaired cognitive capacity in hypoxic conditions.

In addition, the PFC has also been suggested to play a role in the mental fatigue-associated impairment in cognitive performance (Boksem et al. 2006; Boksem and Tops 2008; Pageaux and Lepers 2018; van der Linden and Eling 2006). This is not surprising given the fact that cognitive functions, such as maintaining attention, planning, adaptively changing strategies in the face of negative outcome and ignoring irrelevant information (i.e. being more easily distracted) (Boksem et al. 2006; Boksem and Tops 2008) seem to be impaired after a mentally fatiguing task, and the PFC plays an important role in all of these executive functions. A possible hypothesis is that a decrease in regional cerebral oxygenation, which may be induced by mental fatigue, can alter the PFC-function, and thus cognitive functioning.

Given the fact that both mental fatigue and hypoxia impair cognitive performance and that, in both cases, this impairment appears to be associated with specific changes in brain hemodynamics at the PFC, both stressors (hypoxia and mental fatigue) might interact to further decrease cognitive performance. Therefore, hypoxia, as a mean of decreased oxygen availability, could help understand the mechanisms behind the cognitive performance decrements associated with mental fatigue, which may lead to future countermeasures to prevent the negative effects of mental fatigue. To the best of our knowledge this will be the first study to examine whether the onset of mental fatigue can be manipulated by altering the composition of the ambient air. The main aim of this study was to examine whether oxygenation of the PFC plays a role in the development of mental fatigue and whether acute normobaric hypoxia has an effect on the development of mental fatigue and how this relates to brain oxygenation and hemodynamic responses. We hypothesize that (1) mental fatigue will lead to earlier changes in the prefrontal fNIRS-parameters in combination with lower oxygen availability, (2) that lower oxygen availability will result in earlier onset of mental fatigue, (3) that the effects of mental fatigue on cognitive performance manifest themselves to a greater extent in hypoxic environments.

## Methods

### Participants and study design

Twelve healthy young participants (7 male and 5 female) were included in this study (age 22.9 ± 3.5 years; height 174.2 ± 8.2 cm; mass 69.9 ± 13.9 kg). Participants were excluded if they (1) were younger than 18 years or older than 45 years, (2) had been at altitude (> 2000 m) up to two months before the experiment, (3) were injured and/or used medication at the time of the trial or in two months prior to the trials, (4) were (ex-)smoker and were instructed to (5) withdraw from caffeine 12 h before each trial (6) performing heavy efforts 24 h prior each trial. The experimental procedures and potential risks were explained to the participants and a written informed consent was provided and signed before the start of the study. A randomized, blinded, placebo (PL)-controlled, counter-balanced, cross-over study design was used. Participants were unaware of the goal of this study and were told that the experiment examined the influence of different cognitive tasks and various altitudes on physiological outcome measures such as heart rate and SaO_2_. Participants were blinded for the environmental conditions. After completing all experimental trials, participants were informed about the actual goal of this study. The study protocol was approved by the Ethical Committee of the Brussels University hospital (BUN:. 1,432,022,000,053) and was carried out in accordance with the Declaration of Helsinki.

### Experimental procedure

**Fig. 1:**
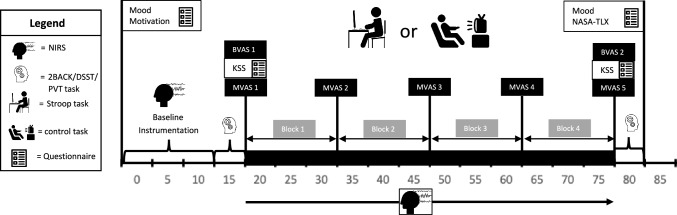
Schematic representation of the experimental and control trial. *fNIRS* functional Near Infrared Spectroscopy; *DSST* Digit Symbol Substitution Task; *PVT* Psychomotor Vigilance Task; *Mood* Brunel Mood Scale; Motivation Matthews Motivation Scale; *BVAS* Boredom Visual Analogue Scale; *MVAS* Mental Fatigue Visual Analogue Scale; *KSS* Karolinska Sleepiness Scale; *NASA-TLX* National Aeronautics and Space Adminstration Task Load Index

The experiment consisted of a familiarization and four consecutive experimental trials, separated by minimum 3 days. All trials were performed in a sound-insulated climate chamber (20 °C and 40% RH) Storex bv, controlled atmosphere, ‘s Gravendeel, The Netherlands) at MFYS at the Vrije Universiteit Brussel. After signing the informed consent, participants completed a familiarization trial in which they performed all procedures as if it was an experimental trial (see Fig. 1), except for the 60-min Stroop task (see cognitive tasks). Instead of the 60-min Stroop task, participants’ maximal cognitive capacity was determined with a maximal Stroop task. The next four experimental trials were performed in a randomized counterbalanced order: (1) mental fatigue (MF) in (normobaric) hypoxia (HYP) (3800 m; fraction of inspired O_2_ (FIO_2_) 13.5%), (2) MF in normoxia (NOR) (98 m; FIO_2_ 21.0%), (3) Control task (CON) in HYP, *(4)* CON in NOR Randomization was assured by using a web-based computer program (www.randomization.com) to determine treatment order (MF_HYP_, MF_NOR_, CON_HYP_ or CON_NOR_ trial; see intervention for details). Participants were blinded from the fraction of inspired oxygen (FIO_2_) and a follow up question (*in which environmental condition do you think you performed today?*) at the end of the experiment confirmed this. During the four experimental trials (see Fig. 1), participants filled in the Brunel Mood Scale, Matthews motivation scale, Karolinska Sleepiness Scale, mental fatigue -Visual Analogue Scale (MVAS) (see psychological measurements) before a 2-min 2BACK task, 90-s DSST-task and a 10-min PVT task (see cognitive tasks). All participants completed the three distinct cognitive tasks in the same order. The 2-BACK task was selected for its ability to assess working memory, the digit symbol substitution test (DSST) was used as a measure of complex scanning or visual tracking and the psychomotor vigilance test (PVT) was chosen as a measure of vigilant attention. Afterwards the 60-min Stroop task (mental fatigue condition) or an emotionally neutral documentary (CON condition) was performed, during which the MVAS was assessed at fixed timepoints. The Stroop task (≥ 30 min.), known for requiring response inhibition and sustained attention, has been shown in prior research to induce mental fatigue *(*Smith et al. 2016; Van Cutsem et al. 2017*).* Finally, the same measurements where repeated with the addition of the National Aeronautics and Space Administration Task Load Index.

### Intervention/CONTROL task

#### Modified stroop task

During the MF-trials, a **modified Stroop task** (Pageaux et al. 2015; Smith et al. 2016), based on their performance during the Stroop Max test (familiarization session) of approximately 60 min, partitioned in 4 blocks of 360 stimuli, was used as the mentally fatiguing task. In this task, four colored words (“red”, “blue”, “green” and “yellow”) were presented one at a time on a computer screen. The participants were required to indicate the color of the word, ignoring the meaning of the word itself. If, however, the ink color was red, the button to be pressed was the button linked to the real meaning of the word, not the ink color. The word presented and its ink color were randomly selected by the computer (100% incongruent), with all incongruent word-color combinations being equally common. Each word was presented on a screen in 34-point font for 1000 ms with an inter-stimulus interval based on their performance on the max test. Participants were instructed to respond as quickly and accurately as possible. To assess performance, ACC and RT were collected and averaged within every block. Each of the 4 blocks were separated by approximately 30 s, during which stimuli kept presenting themselves (20 stimuli), but where the researchers were assessing the subjective feeling of mental fatigue (see psychological parameters). These 20 stimuli where not taken into account for further analysis.

##### Control task

During the control trials, participants had to watch a 60-min emotionally neutral documentary (Our planet, 2019) on the same computer screen that was used for the mental fatigue-trials. To avoid under- and over-arousal and boredom, and to ensure task engagement, the participants had the opportunity to choose between several episodes (One planet, Frozen Worlds, Jungles, Coastal Seas, From Deserts to Grasslands, The High Seas, Fresh Water and Forests) as proposed by the research team. During the control task physiological and psychological measures were assessed at the same time points as during the mental fatigue-trials.

### Cognitive performance test battery

The cognitive tasks were programmed in E-prime 3.0 (Psychology Software Tools, Inc., USA).

#### 2-BACK task

During this test, participants were shown phonetically similar letters (B, C, D, E, G, J, P, T, W), in a randomized order. All letters were presented in white on a black background for 500 ms and with an interstimulus time of 500 ms. Participants had to react correct (green dot/H-key) when the letter that was presented was the same as the letter presented 2 trials before, or incorrect (yellow dot/F-key) when this was not the case. The test lasted 2 min and the outcome measures were RT (Basner et al. 2015) and ACC (Decroix et al. 2018).

##### Digit symbol substitution test (DSST)

During this test, participants were shown a fixated column on the top center of their screen, with 9 numbers, with under each number a symbol. In the center of the screen one of the nine symbols was presented and the aim for the participants was to match the corresponding number as fast as possible. The test lasted 90 s during which the participants had to react to as much symbols as possible. The outcome measures were reaction time (RT) and performance accuracy (ACC) (Decroix et al. 2018).

#### Psychomotor vigilance task (PVT)

During this test, a large white dot (400-point font) appeared on a black background with inter stimulus times varying between 2000 and 10,000 ms (randomized order). This resulted in a total of 100 stimuli being displayed during the 10-min task. Participants had to react by pressing the space bar on a keyboard as fast as possible when the dot appeared. When the space bar was not pressed within 1000 ms after the dot appeared or before the stimulus was presented, this stimulus was classified as “missed”.

### Physiological measures

#### Functional near infrared spectroscopy

fNIRS was measured with the portable OctaMon continuous-wave fNIRS system (Artinis Medical Systems B.V., The Netherlands) and was used to measure relative concentration changes in oxygenated (HbO_2_) and deoxygenated (HHb) hemoglobin concentrations and total hemoglobin (tHb)in the PFC. The 8-channel OctaMon device consists of 8 emitter- and 2 detector optodes, separated by an interoptode distance of 35 mm and functioning at wavelengths of 760 and 850 nm. The OctaMon covered the prefrontal cortical area between Fp1 and Fp2, according to the international EEG 10–20 system (Jasper 1958), and was positioned 10 mm above the participant’s eyebrows. The fNIRS-device was applied after entering the climatic chamber and the measurement started approximately 15 min after entering. A 2-min baseline measure, (resting state) was obtained before the start of the Stroop task or watching the documentary. Afterwards, fNIRS data were collected and averaged during the last 30 s of each block of the Stroop task or corresponding time points during the CON task. fNIRS data was recorded in Oxysoft 3.2.70 (Artinis Medical Systems B.V., The Netherlands) at 10 Hz and markers were placed at baseline (2 min) and at the end of each of the four blocks during the Stroop task or documentary, after which, raw data was exported to MATLAB, within the SPM-fNIRS toolbox. The modified Beer-Lambert law was applied to convert optical densities into relative concentration changes of HbO_2_ and HHb (in μM cm). The age-dependent differential pathlength factor (DPF) (4.99 + 0.067*(Age^0.814^)) was calculated for each participant (Duncan et al. 1996). Next, temporal preprocessing was performed to separately process time series of HbO_2_, HHb and tHb to reduce motion artifacts through the “motion artifact correction (MARA)” function, to reduce cardiac- and respiratory noise and to down sample data to 1 Hz, which is in accordance to Scholkmann et al. (2010). The preprocessed data from the SPM-fNIRS Toolbox was then exported to MATLAB to overplot the markers and extract average 30-s values for (i) baseline, (ii) end of block 1, (iii) end of block 2, (iv) end of block 3 and (v) end of block 4.

##### Oxygen Saturation (SaO_2_)

SaO_2_ was measured during the baseline measurement and during the last 30 s of each block of the Stroop task or documentary by connecting a pulse oximeter with the participant’s right index finger (PULOX ®, Pulsoximeter, CMS50E, Germany).

### Subjective psychological measures

Throughout each experimental trial, multiple subjective outcome measures were collected from the participants. First of all, a pre-test checklist evaluated possible abnormalities before the beginning of each trial, to check if the participant adhered to the mandatory inclusion criteria (e.g., “Did you perform intensive (training) efforts within the last 24 h?”). **Subjective feeling of mental fatigue** was assessed through a visual analog scale (MVAS). Participants were asked to draw a mark on the 10-cm VAS before and after the Stroop task and documentary. During the mental fatigue—and control task, participants where verbally asked the following question: *“from 0 to 100, with zero “not at all”, how mentally fatigued are you right now?”*, after each block of 360 stimuli (Stroop) or corresponding time points (documentary), during which they kept performing the Stroop task of watching the documentary. **Subjective feeling of boredom** was assessed through a visual analog scale (BVAS). Participants were asked to draw a mark on the 10-cm VAS before and after the Stroop task- and documentary, where the far left meant totally not- and the upmost right meant totally bored. **Mood** was assessed through the Brunel Mood Scale (BRUMS) prior as well as post the mental fatigue- and control task. This scale was based on the Profile of Mood States, further details about this validated questionnaire are described by Terry et al. (2003). The success **motivation** and intrinsic motivation scales as described by Matthews et al. (2001) were used to assess motivation before the Stroop task and documentary. **Sleepiness** was assessed through the Karolinska Sleepiness Scale before and after the Stroop task and documentary. This 10-point scale measures the subjective level of sleepiness and goes from 1 = ‘extremely alert’ to 10 = ‘extremely sleepy, falls asleep all the time’ (Kaida et al. 2006). Finally, **subjective workload** was measured with the National Aeronautics and Space Administration Task Load Index (NASA-TLX) (Hart and Staveland (1988) at the end of each trial.

### Statistical analysis

Statistical analysis was conducted through the Statistical Package for the Social Sciences, version 27 (SPSS Inc., Chicago, IL, USA). All data are presented as mean ± standard deviation for *n* = 12 participants, unless stated otherwise. Significance was set at p < 0.05 for all analyses. Normality was assessed with the Shapiro–Wilk test and sphericity was verified by the Mauchly’s test. When the assumption of sphericity was not met, the significance of the F-ratios was adjusted with the Greenhouse–Geisser procedure. For data that were not normally distributed (i.e. sleepiness and “anger” as part of the NASA-TLX) non-parametric Wilcoxon tests were employed to investigate the effect of condition, FIO and/or time. All other parameters [behavioral (Stroop-, 2BACK-, DSST- and PVT RT and ACC), subjective (intrinsic- and success motivation, MVAS, BVAS, mood and NASA-TLX (except “anger”)) and physiological (fNIRS (HbO_2_, HHb and tHb), SaO_2_ and HR] were normally distributed. A 2 × 2 × 5 three-way repeated-measures ANOVA [Mental State (MF, CON) X FIO_2_ (normoxia, hypoxia) X Time (baseline, block 1, block 2, block 3, block4)] was used for MVAS, fNIRS and Stroop task ACC and -RT. For data from the 2BACK, DSST, PVT, SaO_2_ Mood and BVAS, a 2 × 2 × 2 three-way repeated-measures ANOVA [Mental State (MF, CON) * FIO_2_ (normoxia, hypoxia) * Time (pre, post)] was performed. If significant interaction effects were observed, subsequent repeated-measure ANOVA or paired-samples t-tests were performed to elucidate the main effect of condition, FIO_2_ and time. If no significant interaction effects were observed, main effects of condition, FIO_2_ and time were immediately observed and in the case of ‘time’ further interpreted through pairwise comparisons with Bonferroni correction. If no significant interaction effects were observed, main effects of condition and time were immediately observed and further interpreted through pair-wise comparisons with Bonferroni correction.

## Results

### Effect of mental fatigue and FIO_2_ on cognitive performance

#### 2BACK-task

A significant mental state * FIO_2_ (F(1,11) = 21.3, p < 0.001) and FIO * Time (F(1,11) = 6.2, p = 0.030) interaction effect for 2BACK accuracy was found. Within the MF-trials, a significant interaction effect was found within FIO_2_ * time (F(1,11) = 7, p = 0.024). Post hoc paired samples T-test showed that 2BACK ACC in the MF- trials was lower in the post task compared to pre task within the normoxia condition (PRE: 87.1 ± 7.72, POST 75.70 ± 12.88). During the control trials, a significant main effect of FIO_2_ was present (F(1,11) = 12, p = 0.005) only for ACC and not for RT, with lower ACC in the normoxia conditions compared to the hypoxia conditions (NOR: 63.8 ± 5.9; HYP: 79.4 ± 3.7). No other significant effects were observed (see. Table 1)*. See supplementary file for a graphical representation of 2BACK ACC and RT across conditions*.

**Table 1:** Overview of the cognitive outcome measures from the 2BACK-, DSST and PVT task

	Mental Fatigue				Control			
	Normoxia		Hypoxia		Normoxia		Hypoxia	
	PRE	POST	PRE	POST	PRE	POST	PRE	POST
2BACK								
ACC^#^	87.1 ± 7.7^	75.7 ± 12.3	70.3 ± 19.1	70.3 ± 16.6	66.5 ± 21.4	61.2 ± 23.9	75.1 ± 17.7	83.7 ± 10.7
RT (ms)	294 ± 59	274 ± 82	301 ± 44	301 ± 36	294 ± 59	274 ± 82	312 ± 55	323 ± 38
DSST								
ACC	99.1 ± 1	98.4 ± 3.1	97.7 ± 3.5	98.3 ± 2.1	99.1 ± 1.2	99.7 ± 0.8	99.3 ± 2.3	99.3 ± 1
RT (ms)	1449 ± 122	1466 ± 149	1414 ± 159	1444 ± 116	1426 ± 146	1444 ± 163	1409 ± 138	1438 ± 140
PVT								
ACC	95.7 ± 1.7	98.8 ± 11.4	97.4 ± 2.5	99.5 ± 6.5	99.1 ± 0.8	99.3 ± 1.3	97.9 ± 1.3	95.7 ± 3.1
RT (ms)*	333 ± 52	365 ± 50	331 ± 43	364 ± 45	331 ± 38	347 ± 45	335 ± 43	363 ± 71

Data are presented as means ± SD. * denotes a significant main effect of time(p < 0.05). ^ denotes a significant difference between conditions in that specific time interval (p < 0.05). # denotes a significant main effect of FIO_2_

#### DSST-task

No significant differences in DSST RT nor ACC were found between the different times, mental states and hypoxia conditions (see Table 1).

#### PVT

A main effect for time on PVT RT was found (F(1,11) = 19.5, p = 0.001). Independently from mental state and FIO_2_, RT was worse in the post PVT compared to the pre PVT (PRE: 924.54 ± 57.42 s; POST: 995.47 ± 59.89 s) (see Table 1).

#### Stroop task

Both ACC and RT on the Stroop task were not statistically different over time and between hypoxia and normoxia. Furthermore, no additional differences in ACC nor RT were found for the red stimuli (response inhibition) and incongruent stimuli (blue, yellow, green). *See supplementary file for a graphical representation of the effect of hypoxia on Stroop task RT*.

### Effect of mental fatigue and FIO_2_ on prefrontal cortex oxygenation

**Fig. 2:**
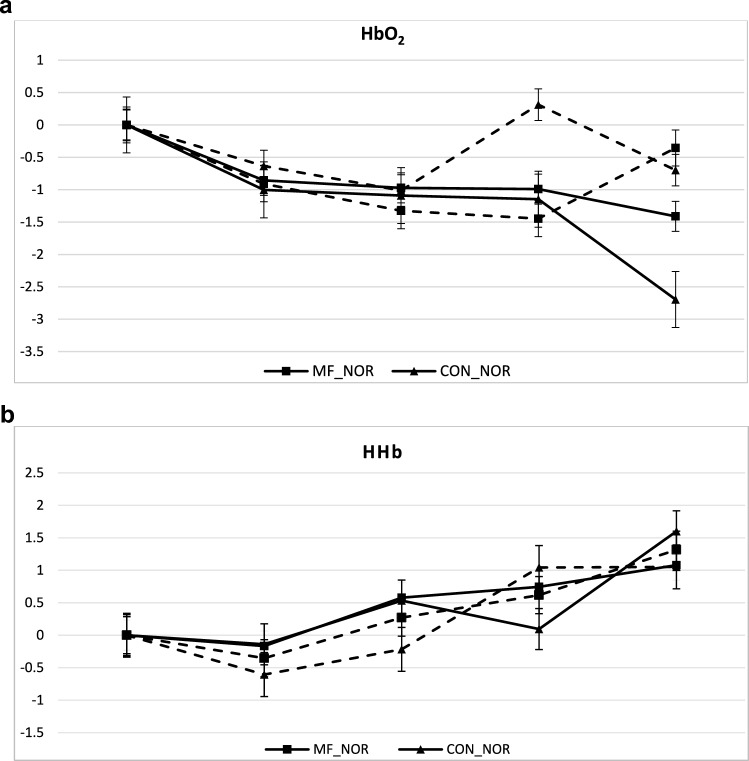
A and 2b Graphs of HbO_2_ and HHb measurements across conditions (graph lines represent means SD)

No effects of time, mental state, nor FIO_2_ were found for HbO_2_ and HHb during the Stroop task or control task (see Fig. 2a, b). For tHb a significant FIO_2_ * mental state * time interaction effect FIO2 (F(4,40) = 0.490, p < 0.047) was present. Post hoc analysis showed that within the normoxia condition there was a greater decrease in tHb at the end of block 2 compared to block 1 (-0.211 1. ± 0.29 mmol VS-0.982 ± 0.354 mmol).

### Secondary outcome measures

#### Psychological measures

**Fig. 3:**
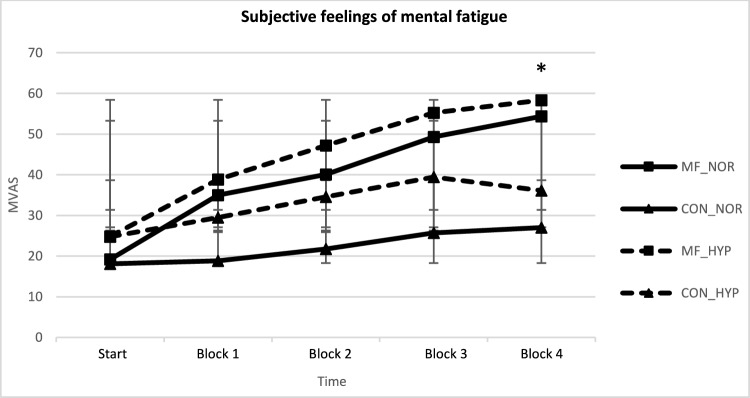
Graph of the MVAS measurements across conditions (graph lines represent means SE; * = significant difference between conditions)

***MVAS.*** FIO_2_ did not interact with any other factor and was observed not to influence the subjective response of mental fatigue on the MVAS. Concerning the effect of time, MVAS significantly increased in time both in MF (F(1.6, 16.2) = 19.3, p < 0.001) and CON (F(1.6, 15.5) = 6.3, p = 0.014). We observed that MF was induced since the MVAS was significantly higher compared to the start of the Stroop task from the third block onwards (p ≤ 0.037; see Fig. 2). In CON no significant differences were observed between the start of the Stroop task and the other time intervals. The subjective feeling of mental fatigue was significantly higher during the Stroop task compared to the documentary at all time points (Block 1: F(1, 10) = 6.4, p = 0.030; Block 2: F(1, 10) = 5.3, p = 0.044; Block 3: F(1, 10) = 8.1, p = 0.018; Block 4: F(1, 10) = 13.4, p = 0.004), except for the start-time interval (see Fig. 3).

***Subjective feeling of boredom*** revealed higher feelings of boredom after the Stroop task compared to the documentary (74 ± 6 VS 32 ± 7.77) F(1,11)32, p < 0.001) and FIO2 was found not to influence boredom.

Both intrinsic and success ***motivation*** were not different between conditions.

Data of ***sleepiness*** was not normally distributed. Therefore, a Friedman test was performed, which was found to be significant (X^2^ = 22.25; p = 0.002). The post hoc Wilcoxon signed rank test found higher sleepiness in the post phase compared to the pre phase for MF_NOR (6 ± 2 VS 4 ± 1), MF_HYP (6 ± 2 VS 4 ± 1) and CON_HYP (6 ± 2 VS 4 ± 2) (p = 0.005).

Regarding the NASA-TLX, it was found that ***frustration*** [F (10,1) = 12.02; p = 0.006] was perceived to be higher after the Stroop task (47.8 ± 6.3 compared to the documentary (30.1 ± 8.1). ***Vigor*** was subject of a FIO_2_ * time interaction effect [F (9,1) = 6.95; p = 0.027]. In the post phase, vgor was significantly higher in the control condition (8.3 ± 3.5) compared to the MF condition (6.3 ± 4.7) in hypoxia. Furthermore, in the MF-condition, vigor was significantly higher in normoxia (9.7 ± 2.7) than in hypoxia (6.6 ± 4.8).

#### Physiological measures

Regarding ***SaO***_***2***_, there was a significant main effect of FIO_2_ (F(1,10) = 220, p < 0.001), with lower SaO_2_ in the hypoxic, compared to the normoxic trials (NOR: 97.9 ± 0.2; HYP: 92.3 ± 0.4%). No effect of mental state nor time was found for SaO_2_. A main effect of time was present for ***blood glucose***, with significantly lower levels in the post phase (end of the trial), compared to the pre phase (beginning of the trial) (89.9 ± 1.2 VS 102.7 ± 3.6 mg/dl). A significant main effect of mental state was present for **HR.** During the Stroop task HR was found to be higher in MF compared to during watching the documentary (77 ± 2 VS 69 ± 3 BPM) (F(1.11) = 19.879 P < 0.001). Additionally, a main effect of time resulted in HR to be lower at the end of each block compared to baseline (F(1.11) = 7.921 p < 0.001), but no differences in HR were present between the end of the four blocks. FIO_2_ was found not to influence HR.

## Discussion

The results of the present study suggest that FIO_2_ does not impact subjective feelings of mental fatigue. In agreement with previous research (Van Cutsem et al. 2017), we can confirm that performing a 60-min Stroop task at the participants’ maximal cognitive capacity results in higher subjective feelings of mental fatigue compared to watching an emotionally neutral documentary (Van Cutsem et al. 2017). Meanwhile, the oxygenation of the PFC was not affected by either the difference in FIO_2_, nor by the participants’ mental state.

### Effect of FIO_2_ on self-reported mental fatigue and cognitive performance

We hypothesized that through the increased cerebral metabolic demand, hypoxia would result in an earlier onset of mental fatigue, and that the effects of mental fatigue on cognitive performance would manifest themselves to a greater extent with lower oxygen availability. However, this study found no evidence for either of these hypotheses. The individualized Stroop task successfully induced mental fatigue, which can be seen in the increased MVAS values at the end of each block during the Stroop task compared to the emotionally neutral documentary. This is in line with previous research, which states that prolonged periods of intense cognitive activity result in increased subjective feelings of mental fatigue (Marcora et al. 2009; Van Cutsem et al. 2017). Although we assumed that hypoxia would impact mental fatigue, results show that the hypoxia condition did not alter the subjective feelings of mental fatigue, as there was no difference in MVAS during the Stroop task in normoxia compared to in hypoxia, nor was there a difference in MVAS during watching the emotionally neutral documentary in normoxia compared to in hypoxia. A potential reason for this finding not being statistically significant is the small sample size, since a trend to significance was present.

Regarding cognitive performance, no changes were found for the DSST nor the PVT-task. However, there was a lower ACC on the 2BACK task in the post task compared to the pre task in both MF_NOR and CON_NOR. A lower ACC in the post task when one is mentally fatigued is similar to previous research that states that mental fatigue has a negative effect on cognitive performance (Tanaka 2015; Van Cutsem et al. 2017; Wascher et al. 2014). However, in hypoxia, this decrease in cognitive performance was not present. This is surprising since hypoxia, in this case 13.5% O_2_ (3800 m), is known to negatively alter cognitive performance (Martin et al. 2019; Ochi et al. 2022). The impairment in ACC on the 2BACK task, observed in post task for the control condition (CON_NOR) could potentially be attributed to several factors. In the control condition participants were not subjected to the same cognitive paradigm as during the mental fatigue condition, however they still slightly experienced cognitive demands throughout the experimental protocol. This could have contributed to a certain degree of mental fatigue, albeit to a lesser extent than in the mental fatigue condition. Additionally, factors such as boredom and monotony may have influenced performance. Participants might have experienced similar psychological states or fatigue-related effects due to the duration of the experimental protocol in the control condition. Recently, O’Keeffe et al. (2021) investigated the individual and combined effects of mental fatigue and hypoxia on physical and cognitive performance and found no effect of mental fatigue or hypoxia on cognitive performance, reinforcing our results. In their study, participants completed six experimental trials of which three in a mentally fatigued state and three control trials, both in normoxia (20.9% O_2_), mild-hypoxia (13% O_2_) and severe hypoxia (10%O_2_). Neither mental fatigue, nor hypoxia influenced the performance on the Tower of Hanoi-task (Humes et al. 1997), which is, like the 2BACK-task, a measure of working memory and is meant to assess prefrontal cortex related cognitive functions, including planning abilities. O’Keeffe et al. (2021) discussed multiple potential mechanisms/reasons for the null effect, such as the complexity of the cognitive task and the duration of the exposure to hypoxia. The complexity of the Tower of Hanoi- and the 2BACK task makes it susceptible for learning effects and thereby, it is found that the number of training sessions is positively related to the size of transfer effect of working memory (Soveri et al. 2017). Training and repeated execution of the task could lead to the generation of performance strategies, improvement in monitoring and updating of working memory content and improved inhibition of items that are no longer relevant (Pahor et al. 2022). Therefore, due to the fact that in our study participants performed the task two times per trial over the count of four separate trials, learning effects could have interfered with the results and therefore result in the null effect of mental fatigue and hypoxia (Soveri et al. 2017).

Another possible explanation for the null effect of mental fatigue and hypoxia is the acclimation period. In our study, participants immediately entered the climatic chamber upon arrival, which was already set at the desired altitude. Previous studies have shown that hypoxia already negatively influences cognitive performance in durations as short as 30-min performance (Crow and Kelman 1971; Loprinzi et al. 2019; Lowe et al. 2007). Since the acclimation period took about 20 min in our study, it is possible that cognitive performance was already decreased before any tests were performed. As we did not diminish the amount of oxygen any further during the trial, no further changes in cognitive performance could have been detected in the present study. Unfortunately, in the present study, no measures were included to substantiate this speculation. Some other studies also found no effect of acute hypoxia on performance of the 2-back task and the Stroop task (Bouak et al. 2018; Crow and Kelman 1971; Legg et al. 2016). Crow and Kelman, (1971) argued that mild hypoxia could result in a relatively selective defect of psychological function, for example, the capacity to acquire a strategy (i.e. long-term learning) for dealing with a complex information-processing task to be impaired at levels of hypoxia at which other functions remain unaffected. A possibility is that different aspects of cognitive function may be more prone to impairments at different degrees of hypoxia, in turn adding to a diversity of results across studies linking hypoxia to cognition (Bouak et al. 2018; Legg et al. 2016).

### Prefrontal cortex oxygenation during the Stroop task and the effect of hypoxia

We hypothesized that mental fatigue would lead to earlier changes in prefrontal fNIRS-parameters with lower O_2_ availability. Contrary to our expectations, no differences in HbO_2_ and HHb were found during and between the execution of the 60-min Stroop task or during the emotionally neutral documentary. A prolonged (3 h), simulated driving task in the study of Li et al. (2009) resulted in increased feelings of tiredness, laziness, irritability, lack of energy and sluggishness. Li et al (2009) reported a steady state in fNIRS oxygenation parameters after an initial increase of HbO_2_ at the start of the task, until approximately min 110, after which HbO_2_ levels dropped until the end. We cannot confirm our hypothesis, since no changes HbO_2_ nor HHb were present. This finding could be attributed to a time-on-task effect. The duration of the Stroop task (60 min) may have been insufficient to induce significant mental fatigue or changes in PFC oxygenation. In contrast, Li et al (2009) reported PFC deoxygenation starting around min 110 during a prolonged simulated car driving task, suggesting that fatigue-related changes may occur over longer durations. Another explanation could be the use of different mental fatigue-inducing paradigms (Stroop task vs. simulated driving task), which likely engage distinct cognitive processes and neural mechanisms. Therefore, the Stroop task may not have effectively induced mental fatigue or affected PFC oxygenation compared to the simulated car driving task used in previous studies (Massar et al. 2010).

The NBACK, DSST and PVT tasks are all cognitive tasks which require activation of the PFC (Basner et al. 2015; Decroix et al. 2018). A systematic review by Bonetti et al. (2019) indicated that working memory tasks (e.g. the N-BACK) elicit increases in HbO_2_ in the early phases of the task. Moreover, Causse et al. (2017a, 2017b), reported greater increases in HbO_2_ when the difficulty of the task was higher (2BACK-1BACK-0BACK), and Csipo et al. (2021) reported that more demanding cognitive tasks evoked a greater neurovascular coupling response. Previous studies thus prove that oxygenation of the PFC increases in the initial stages of cognitive activity. The direct succession of the Stoop task to the three cognitive tasks (2BACK, DSST, PVT) could be the reason why no increase in oxygenation was found at the onset of the 60-min Stroop task. The initial increase in PFC oxygenation possibly already occurred during the cognitive tasks and was thus not measured, since the aim was to examine PFC-oxygenation throughout the mentally fatiguing and control tasks.

Regarding the effect of hypoxia on prefrontal fNIRS, we hypothesized that mental fatigue would lead to earlier changes in the prefrontal fNIRS-parameters in combination with lower oxygen availability. Although peripheral SaO_2_, measured at the participant’s left index finger, was lower in the hypoxic conditions compared to normoxia, hypoxia had no effect on the cognitive task induced changes in PFC-oxygenation. Thus, the lower availability of oxygen in the hypoxic condition did not influence cerebral oxygen delivery. A potential reason for the null effect of hypoxia could be that by the time the fNIRS-measurements started, the lower FIO_2_ had already affected the PFC, causing no further changes within the hypoxic conditions (Crow and Kelman 1971; Loprinzi et al. 2019; Lowe et al. 2007). However, as previously stated, no measures were included in the present study to substantiate this speculation and further research is needed.

## Limitations and future research

A potential limitation of the study was that the Stroop-task/documentary immediately followed the completion of the cognitive test battery and that fNIRS was not continuously measured throughout the entire experiment. These tasks prior to the intervention could have already induced early oxygenation changes to the PFC, thereby distorting the effects of the 60-min Stroop-task/documentary. Future studies should provide a sufficient resting period before measuring physiological brain parameters. Although we did not find any statistical difference indicating that oxygenation of the PFC plays a role in the development of mental fatigue, further research on this topic is warranted, with a larger pool of participants to increase the power of the study. Transcranial direct current stimulation (tDCS) could be a potential intervention to modulate oxygen levels (Figeys et al. 2021). First of all, future studies need to include lateralization and distinct subregions of the PFC within their measuring methods. Previously, a fMRI study provided evidence that the dorsolateral PFC during the Stroop task plays a predominant role in the top-down attention control (Milham et al. 2003). This study showed that (1) the cognitive tests prior to the Stroop task may have already affected PFC oxygenation and (2) that distinct regions within the PFC require more resources (e.g. oxygen) while others may be diminished. Measuring the PFC in its entirety may have negated the effects of mental fatigue on distinct regions of interest within the PFC. Finally, future studies should elaborate on the acute effects of ascending to altitude or decreasing levels of PaO_2_ on PFC oxygenation and the build up to mental fatigue as this could contribute to a possible minimal supply of oxygenation to the PFC.

## Conclusion

This is the first study to investigate the role of PFC-oxygenation during the build up to mental fatigue and the associated interaction effects with altered oxygen availability. Although we successfully induced mental fatigue, normobaric hypoxia seemed to have no effect on the severity of self-reported mental fatigue and subsequent cognitive function. Additionally, this study could not provide evidence for oxygenation of the PFC to play a role during a mentally fatiguing task.

### Supplementary Information

Below is the link to the electronic supplementary material.

Supplementary file1 (DOCX 50 KB)

## Data Availability

The data that support the findings of this study are available from Vrije Universiteit Brussel upon reasonable request.
