# Supplementary material for: Prefrontal cortex oxygenation during a mentally fatiguing task in normoxia and hypoxia
Source: Exp Brain Res. 2024 Jun 5;242(7):1807–19. doi: 10.1007/s00221-024-06867-y (PMC11208267; doi:10.1007/s00221-024-06867-y)
Supplement: Supplementary file 1 — Supplementary file1 (DOCX 50 KB) [file 221_2024_6867_MOESM1_ESM.docx]

**Supplementary material:**

**
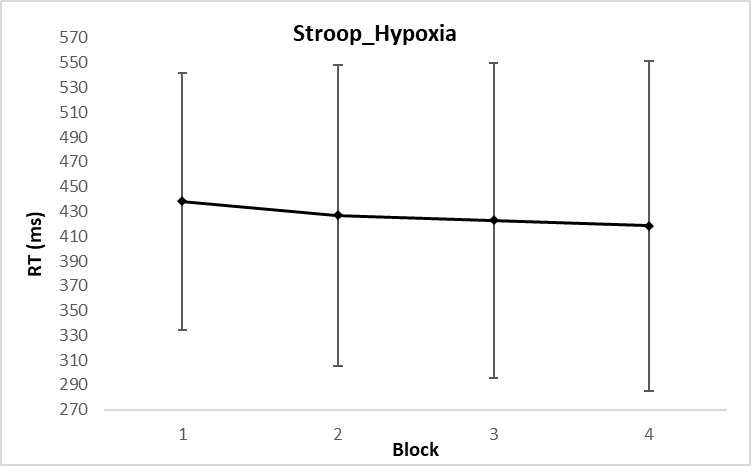
**

**
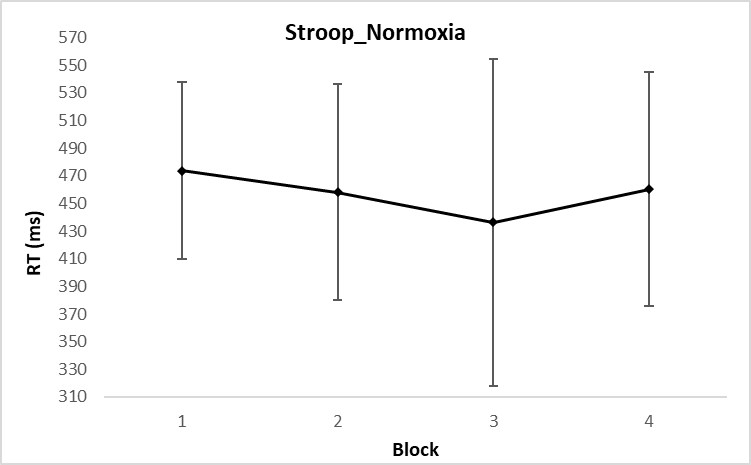
**

*Figure 4a and 4b Graphs of Stroop task RT in hypoxia and normoxia (graph lines represent means SD).*

**
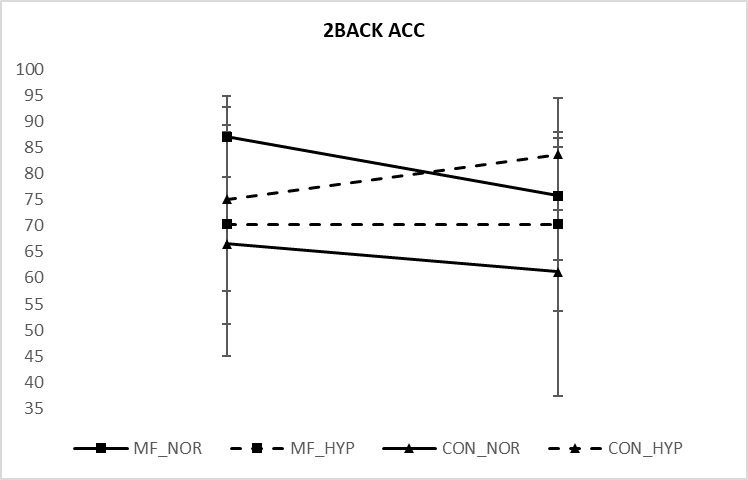
**

**
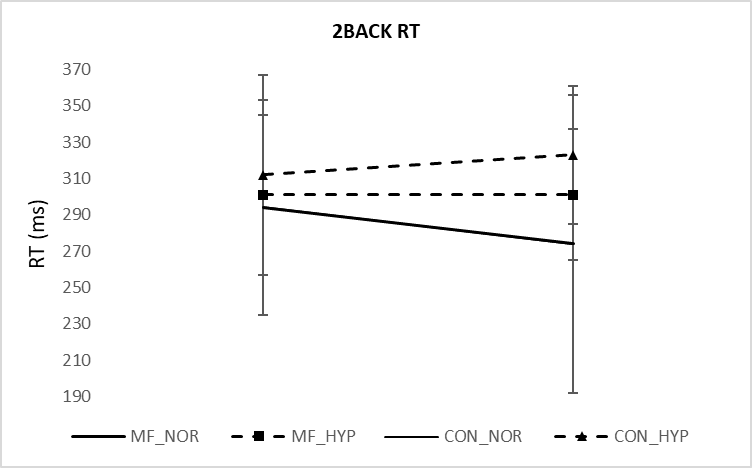
**

*Figure 5a and 5b Graphs of 2BACK ACC and RT across conditions (graph lines represent means SD).*
